# Surgical, pathological, and microbiological characterization of cutaneous keloid fibroma in the thigh region of dromedary camels (*Camelus dromedarius*): nine cases (September 2022–April 2026)

**DOI:** 10.3389/fvets.2026.1886183

**Published:** 2026-08-10

**Authors:** Madeh Sadan, Walid Refaai, Fahd Al-Sobayil, Elhassan M. A. Saeed, Yousef M. Alharbi, Khalid S. Almushawwah, Abdulrahman A. Alkheraif, Mohie Haridy

**Affiliations:** 1 Department of Clinical Sciences, College of Veterinary Medicine, Qassim University, Buraydah, Saudi Arabia; 2 Department of Surgery, Anesthesiology, and Radiology, Faculty of Veterinary Medicine, Zagazig University, Zagazig, El Sharkia, Egypt; 3 Department of Pathology and Laboratory Diagnosis, College of Veterinary Medicine, Qassim University, Buraydah, Saudi Arabia; 4 Department of Medical Biosciences, College of Veterinary Medicine, Qassim University, Buraydah, Saudi Arabia

**Keywords:** animals, cutaneous keloid fibroma, pathology, pathophysiology, *Staphylococcus warneri*, thigh region

## Abstract

**Aim:**

This study aimed to investigate the clinical, microbiological, and histopathological characteristics of cutaneous keloid fibroma and to evaluate the efficacy of surgical management in camels.

**Methods:**

Nine dromedary camels of various breeds (five Asfar, two Shageh, one Wadeh, and one Mejhem) presented with cutaneous fibrous masses located at the caudal aspect of the thigh region. All cases underwent detailed clinical assessment followed by microbiological and histopathological analyses.

**Results:**

Clinically, lesions were characterized by firm, well-demarcated, and slowly progressive masses; all cases (9/9) were non-painful on palpation, while restricted mobility and functional impairment were observed in a subset of cases (noted in larger or strategically located lesions). Microbiological examination revealed *Staphylococcus warneri* in all examined cases (9/9), indicating consistent secondary bacterial colonization rather than a primary diagnostic feature. Histopathological findings demonstrated marked fibro-collagenous proliferation with dense hyalinized collagen bundles, low cellularity, and minimal inflammatory infiltration, consistent with keloid fibroma. Surgical excision with complete removal of the fibrous mass was performed in all cases and was found to be safe and effective. Postoperative outcomes were favorable, with satisfactory wound healing and no evidence of recurrence during a six-month follow-up period.

**Conclusion:**

Clinical and histopathological evaluations are essential and sufficient for accurate diagnosis, whereas microbiological findings support identification of secondary infection but are not indispensable for diagnosis. Surgical excision remains the treatment of choice, providing favorable clinical outcomes and restoration of function, with consistent terminology aligned with the reported results.

## Introduction

Dromedary camels (*Camelus dromedarius*) represent a major livestock species in arid and semi-arid regions, particularly in Saudi Arabia and other parts of the Middle East and North Africa, contributing substantially to meat and milk production, transportation, and socio-economic stability (1, 2). The overall health status of camels is a critical determinant of productivity, product quality, and reproductive performance. Cutaneous disorders, particularly those affecting functional anatomical regions such as the thigh, can adversely compromise animal welfare and production efficiency (3, 4). Among these conditions, fibrous proliferative lesions including fibroma, constitute a clinically relevant yet underreported entity in camels (5, 6). Keloid fibroma is characterized by excessive fibroblast proliferation and abnormal collagen deposition following dermal injury, resulting in the formation of firm, slowly enlarging masses (7). Although considered benign and non-neoplastic, its clinical features may resemble neoplastic or chronic inflammatory conditions, complicating accurate diagnosis and therapeutic decision-making (7, 8).

In human medicine, keloid and keloid-like lesions are well documented and represent an abnormal wound-healing response marked by persistent fibroblast activation and excessive deposition of disorganized, hyalinized collagen. These lesions commonly develop after trauma, surgical procedures, burns, or inflammatory skin disorders and are often associated with genetic susceptibility and dysregulated cytokine expression. Clinically, human keloids present as firm, raised scars that may be accompanied by pruritus or discomfort and frequently recur following surgical excision (9, 10). In veterinary medicine, similar fibrous proliferative lesions have been reported in dogs, although they are relatively uncommon and are variably described as fibroma, collagenous hamartoma, or keloid-like lesions. These lesions typically arise secondary to trauma and present as well-circumscribed, firm cutaneous masses. Histologically, they resemble human keloids, with dense collagen bundles, variable cellularity, and minimal cellular atypia. Despite their proliferative appearance, these lesions are benign and lack metastatic potential (8, 11).

The pathogenesis of keloid fibroma is often linked to prior trauma, surgical intervention, chronic abscessation, or persistent irritation, all of which may disrupt skin integrity and facilitate microbial invasion. Secondary bacterial colonization may further influence lesion development and persistence. Bacterial species commonly associated with cutaneous lesions in camels include *Corynebacterium pseudotuberculosis, Corynebacterium ulcerans, Trueperella pyogenes, Streptococcus* spp.*, and Staphylococcus* spp. (12, 13). These microorganisms can contribute to chronic inflammation and structural tissue changes. Pathogens typically gain entry through breaches in the skin or mucosal surfaces or may be introduced into deeper tissues via contaminated foreign materials (14).

Clinically, keloid fibroma presents as a well-demarcated, firm, non-painful swelling with slow progression and with no inflammatory signs. Histopathologically, it is characterized by dense, hyalinized collagen bundles with low cellularity and minimal mitotic activity (15, 16). Accurate diagnosis requires an integrated approach that combines clinical evaluation with complementary diagnostic methods, including ultrasonography, microbiological analysis, and histopathological examination. Surgical excision is the primary treatment modality and provides definitive histopathological confirmation, especially when the lesion’s size or location affects normal function; however, an initial diagnosis may also be established through biopsy (3, 4, 17–20).

The present study aims to characterize the clinical, ultrasonographic, microbiological, and histopathological features of cutaneous keloid fibroma in dromedary camels, with particular emphasis on lesions affecting functionally important anatomical regions. We hypothesize that keloid fibroma in camels represents a benign but distinct fibroproliferative condition associated with prior tissue injury and possible secondary microbial involvement, and that an integrated diagnostic approach can reliably differentiate it from neoplastic and other chronic inflammatory lesions.

## Materials and methods

### Animals and case selection

Nine adult dromedary camels (8 males and 1 female) were presented to the University Veterinary Hospital, Faculty of Veterinary Medicine, Qassim University, Saudi Arabia, between September 2022 and April 2026. Their ages ranged from 50 to 180 months (mean ± SD: 95 ± 12 months), and body weights ranged from 450 to 700 kg (mean ± SD: 470 ± 140 kg). The animals represented different breeds, including Asfar (*n* = 5), Shageh (*n* = 2), Wadeh (*n* = 1), and Mejhem (*n* = 1).

**Figure 1:**
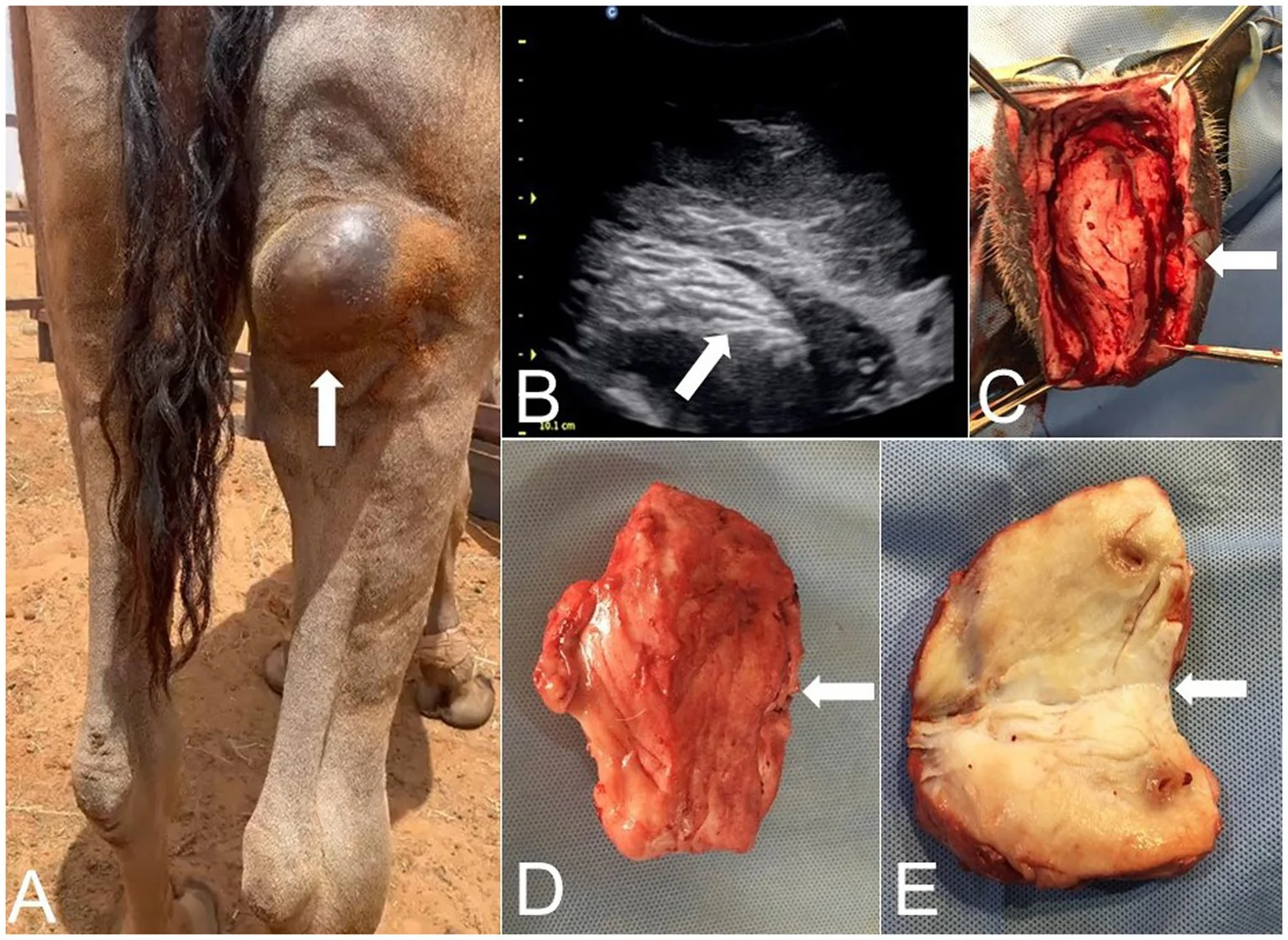
**(A)** Cutaneous keloid fibroma (13 × 11 × 7 cm) at the caudal aspect of the thigh region in Asfar camel (arrow). **(B)** Ultrasonographic image showing heterogeneous and hypoechoic to isoechoic contents with thick internal septa enveloped in a thick echogenic capsule (arrow). **(C)** Operative image for resection of the cutaneous keloid fibroma (arrow). **(D)** Post-operative image of the resected tissue revealed whitish tan mass, firm in consistency and poor in vasculature. **(E)** Cut section showed poor vascularization and whitish tan colour of the resected keloid fibroma mass.

**Figure 2:**
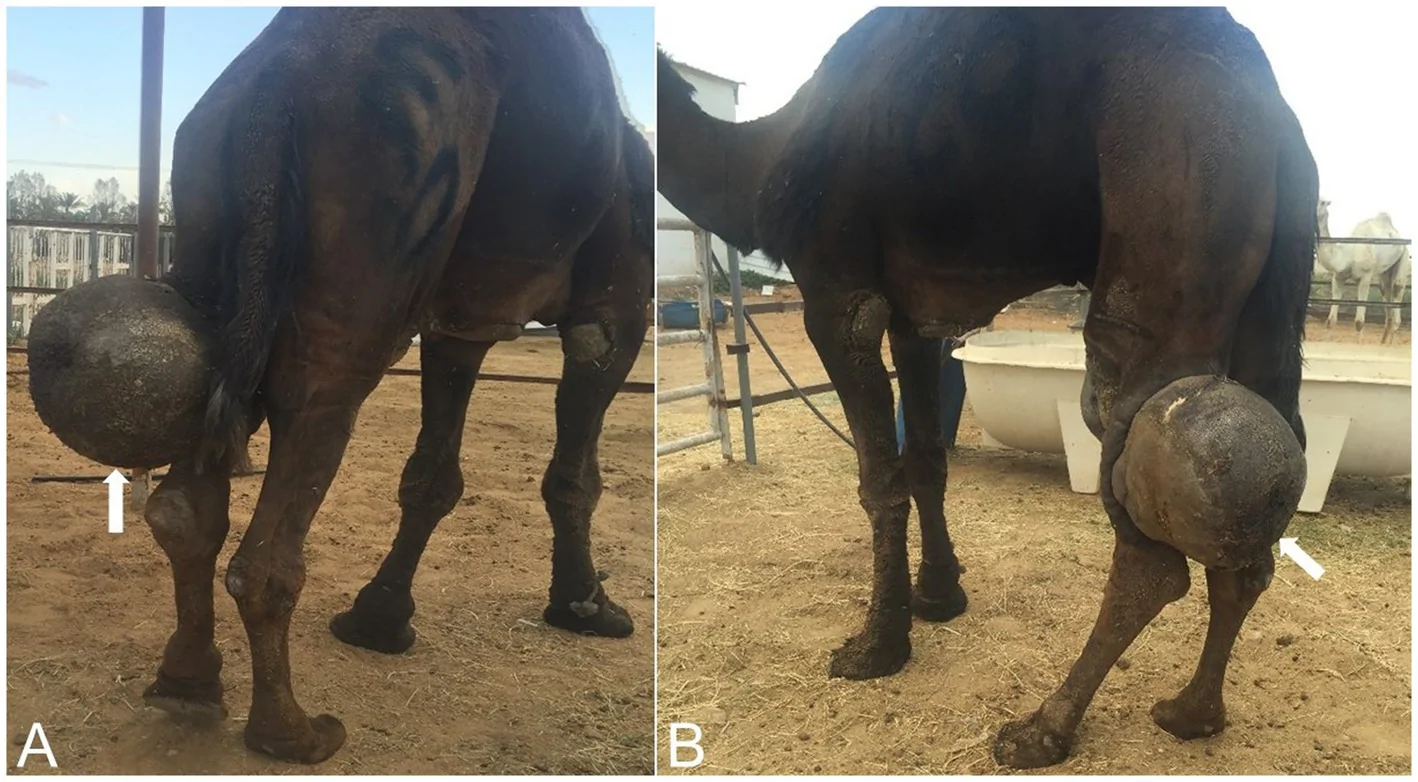
**(A,B)** Huge cutaneous keloid fibroma (50 × 49 × 47 cm) at the caudal aspect of the thigh region extended to the leg region in a male Mejhem camel (arrows).

**Figure 3:**
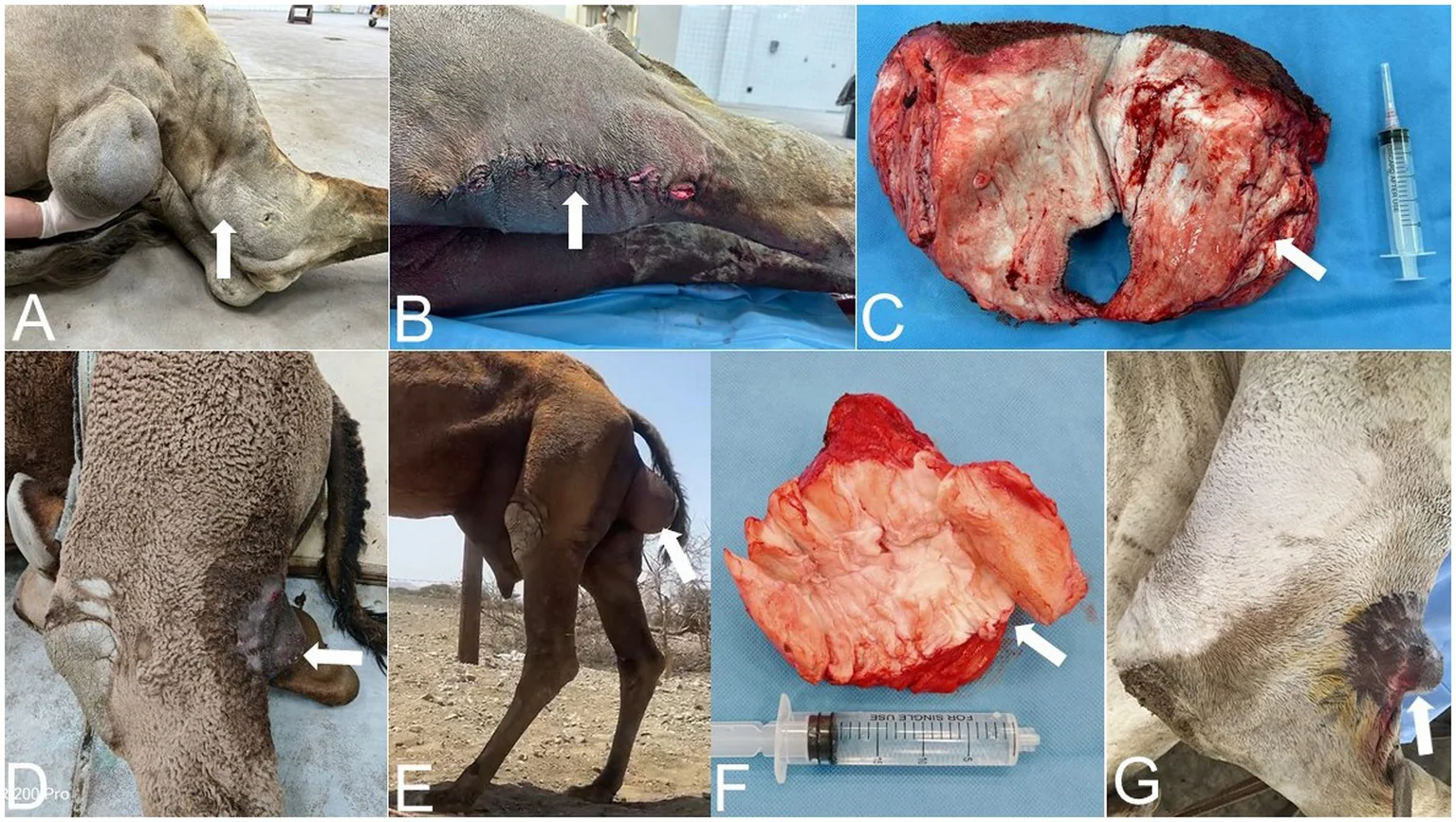
**(A)** Cutaneous keloid fibroma (approximately 23 × 19 × 11 cm) at the caudal aspect of the thigh region in male Asfar camel (arrow). **(B)** Skin closure after resection of keloid in the same case. **(C)** Post-operative image of the resected keloid fibroma revealed avascular whitish tan firm mass. **(D)** Cutaneous keloid fibroma (approximately 11 × 8 × 5 cm) at the caudal aspect of the thigh region in male Asfar camel (arrow). **(E)** Huge cutaneous keloid fibroma (approximately 33 × 20 × 16 cm) at the upper caudal aspect of the thigh region in male Asfar camel (arrow). **(F)** Post-operative image of the resected keloid fibroma in case (D). **(G)** Cutaneous keloid fibroma (approximately 12 × 9 × 6 cm) at the caudal aspect of the thigh region in male Wadeh camel (arrow).

**Figure 4:**
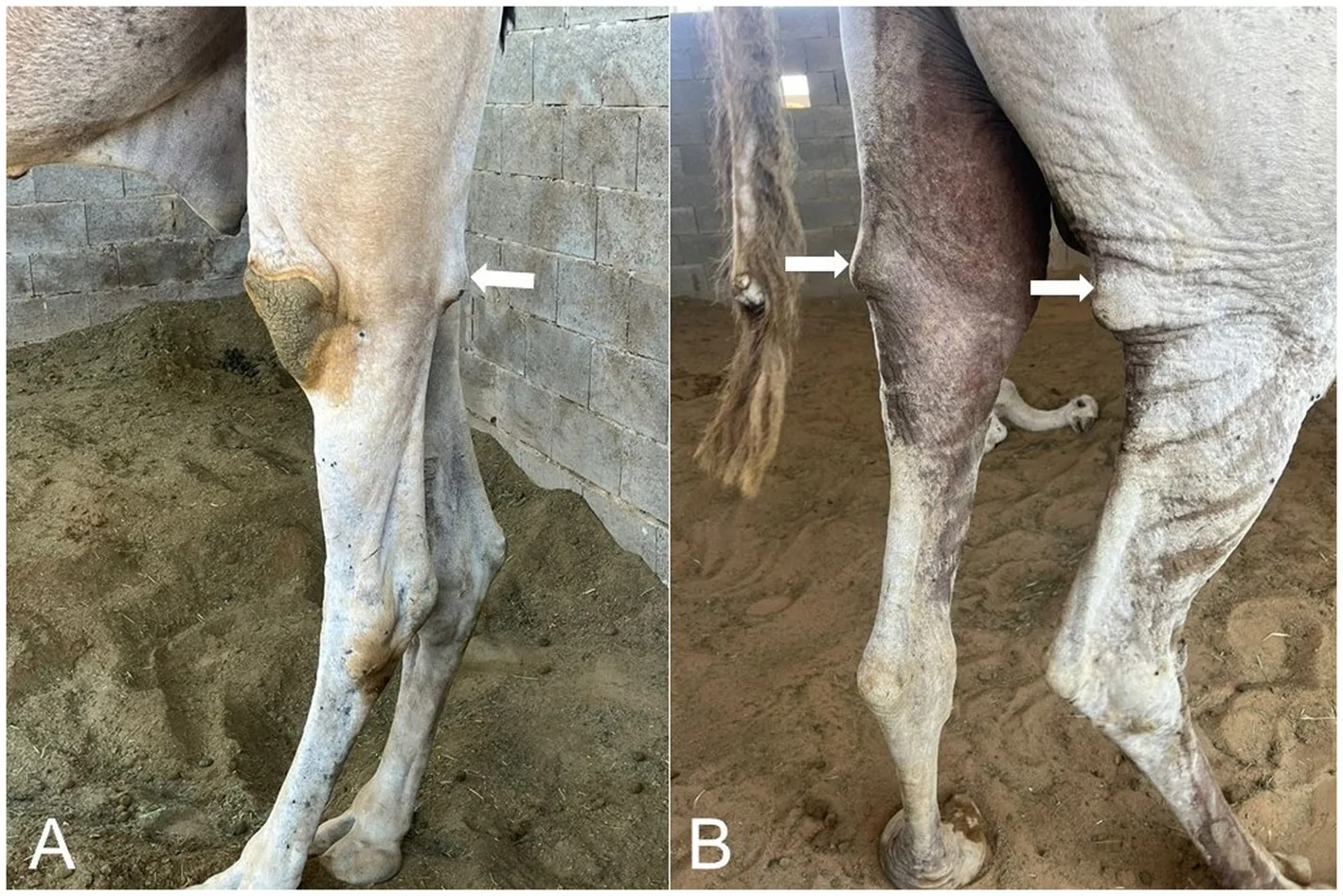
Cutaneous keloid fibroma (approximately 4 × 3 × 2.5 cm) at the caudal aspect of the thigh region in Shageh male camel (**A**, arrow). Bilateral cutaneous keloid fibroma (approximately 4 × 3 × 3 cm) at the caudal aspect of the thigh region in Shageh female camel (**B**, arrows).

**Figure 5:**
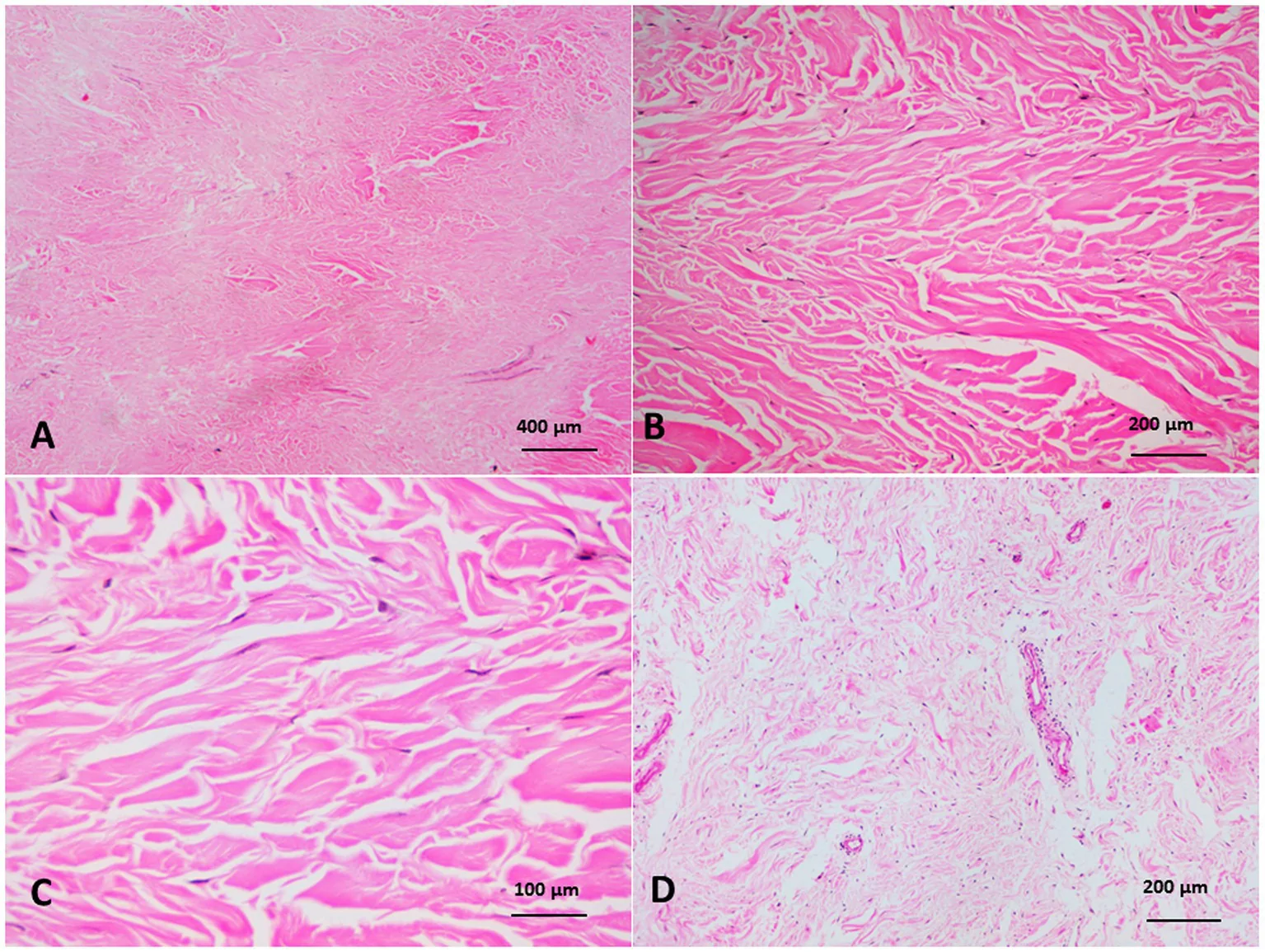
**(A)** Keloid fibromas were mostly composed of abundant bundles of thick hyalinized collagen fibers contained (H&E, bar = 400 μm) **(B,C)** scarcely fusiform fibrocytes and rarely macrophages and lymphocytes around blood vessels (H&E, B: bar = 200 μm, C: bar = 200 μm) **(D)** The lesions were less dense and more cellular at their periphery than at their center and the periphery is more rich in inflammatory cells (H&E, bar = 200 μm).

**Figure 6:**
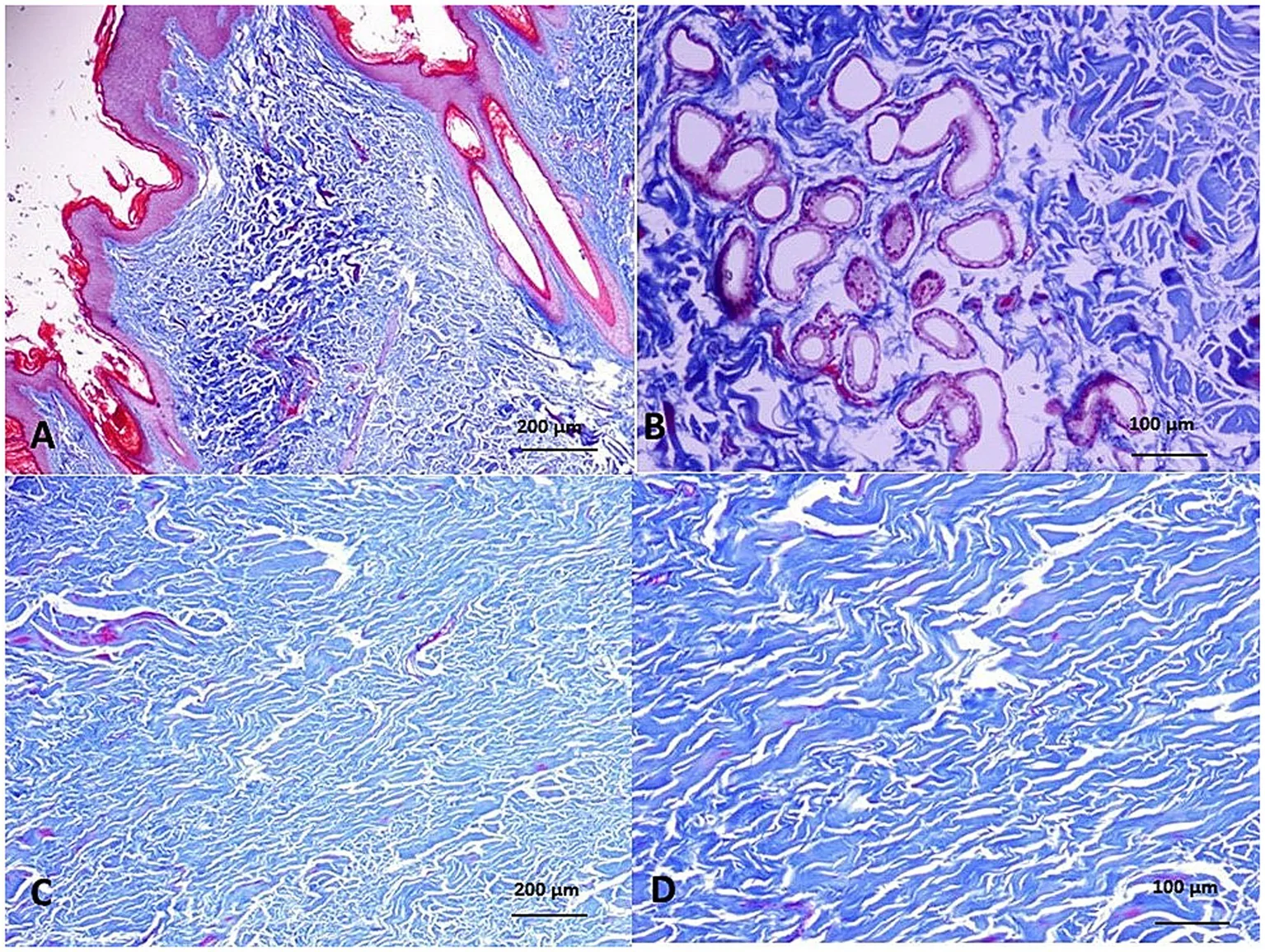
**(A)** Hyalinized collagen fibers were predominantly located at the center of the lesion but extend even in the subepidermal layer (Masson’s trichrome, bar = 200 μm), **(B)** pressing on sweat glands and hair follicles (Masson’s trichrome, bar = 100 μm). **(C)** Absence of neovascularization and granulation tissue (Masson’s trichrome, bar = 200 μm). **(D)** Most of component of keloid fibromas were mature hyalinized collagenous fibers with scant fibrocytes (Masson’s trichrome, bar = 100 μm).

Cases were selected based on clinical presentation, including chronic, firm caudal thigh masses consistent with keloid fibroma, supported by microbiological and histopathological findings (Figures 1–6). The study protocol was approved by the Animal Welfare and Ethics Committee of Qassim University (Approval No. 367).

### Clinical and ultrasonographic evaluation

All camels underwent thorough clinical examination to assess lesion characteristics and duration. Signalmen data (age, sex, and breed) were recorded and summarized (Table 1). Ultrasonographic examination was performed with animals in lateral recumbency using a real-time ultrasound machine (SSD 500, Aloka, Tokyo, Japan) equipped with 2.5–5.0 MHz sector and 7.5–10.0 MHz linear transducers. The affected region was clipped, shaved, and prepared with coupling gel. Ultrasonography was used to evaluate lesion size, wall thickness, echogenicity, internal architecture, and contents. When indicated, ultrasound-guided aspiration was performed under light sedation using intravenous xylazine hydrochloride (0.2 mg/kg) to obtain samples for microbiological analysis. All examinations were conducted by a single experienced operator to ensure consistency.

**Table 1:** Clinical findings of cutaneous keloid fibroma in thigh region in camels (*Camelus dromedarius*) (*n* = 9).

Case No.	*Age (Yrs)	Breed	Sex	Duration of lesion/per month	Approximate size	Precise location
1	6	Asfar	Male	3 months	13 × 11× 7 cm	At the caudal aspect of the thigh region
2	8	Wadeh	Male	5 months	12 × 9 × 6 cm	At the caudal aspect of the thigh region
3	9	Asfar	Male	3 months	23 × 19 × 11 cm	At the caudal aspect of the thigh region
4	5	Asfar	Male	4 months	11 × 8 × 5 cm	At the caudal aspect of the thigh
5	15	Asfar	Male	3 months	13 × 9 × 8 cm	At the caudal aspect of the thigh
6	10	Mejhem	Male	6 months	50 × 49 × 47 cm	At the caudal aspect of the thigh region extended to the leg region
7	6	Shageh	Male	8 months	4 × 3 × 2.5 cm	At the caudal aspect of the thigh
8	7	Shageh	Female	1 year	4 × 3 × 3 cm	At the caudal aspect of the thigh
9	5	Asfar	Male	3 years	33 × 20 ×16 cm	At the upper caudal aspect of the thigh

### Pre-operative management

All camels underwent pre-operative clinical assessment to confirm suitability for sedation and surgical intervention. Stabilization included fluid therapy and control of pain and infection. Intravenous fluids consisted of 0.9% normal saline administered at a rate of 5–10 mL/kg/h (Saudi Pharmaceutical Solution Industry, Riyadh, Saudi Arabia), adjusted according to the animal’s clinical condition. Intravenous administration of 5% dextrose solution was provided at a rate of 2–5 mL/kg/h (Saudi Pharmaceutical Solution Industry, Riyadh, Saudi Arabia). Antimicrobial therapy included intramuscular procaine penicillin G (30,000 IU/kg) and streptomycin (10 mg/kg; Norbrook Laboratories, UK). Analgesia and anti-inflammatory treatment were provided using flunixin meglumine (1.1 mg/kg; Finadyin, Schering-Plough, UK). Sedation was achieved with intravenous xylazine hydrochloride (0.3 mg/kg; Norbrook Laboratories, Newry, UK).

### Surgical management (intra-operative procedure)

Animals were restrained in lateral recumbency, and the affected area was clipped and aseptically prepared. Local infiltration anesthesia was achieved using 2% lidocaine hydrochloride (Norbrook Laboratories, UK) administered circumferentially around the lesion. A wide elliptical skin incision was made over the keloid fibroma, including the fibrotic capsule to ensure complete excision. Hemostasis was achieved using ligation and, where necessary, interrupted horizontal mattress sutures with absorbable suture material (polyglactin 910, No. 2). The surgical field was irrigated thoroughly with sterile saline followed by an antiseptic solution. The wound was closed using horizontal mattress sutures. A passive drain was placed in selected cases with large surgical dead space, extensive tissue dissection, or anticipated postoperative exudate accumulation.

### Post-operative management and follow-up

Post-operative care focused on infection control, inflammation reduction, and wound healing. The antimicrobial and anti-inflammatory treatments initiated pre-operatively were continued for five days. Additionally, camels received intramuscular vitamin AD₃E (10 mL) to support recovery. Animals were housed under stall confinement for approximately one month to limit movement and reduce wound contamination. Daily monitoring included assessment of wound healing, swelling, exudate, and general health status. Wounds were managed through routine cleaning and antiseptic dressing, and monitored for complications such as infection or dehiscence. Drains, when used, were removed once exudation subsided.

Skin sutures were removed 10–14 days post-operatively, depending on healing progress. Animals were considered clinically recovered after approximately four weeks, based on satisfactory healing and absence of complications. Long-term outcomes were assessed six months post-operatively through owner follow-up to evaluate recurrence, discharge, or functional impairment, confirming favorable healing and absence of recurrence.

### Microbiological examination

Pus and tissue samples were aseptically collected before and during surgery and transported under chilled conditions. Samples were cultured on sheep blood agar and incubated aerobically at 37 °C for 24–48 h. Bacterial identification was based on colony morphology, hemolytic pattern, Gram staining, catalase and coagulase tests. Suspected staphylococcal isolates were further identified and subjected to antimicrobial susceptibility testing using the VITEK 2 Compact system (bioMérieux, France).

### Histopathological examination

Excised keloid tissue samples were fixed in 10% neutral buffered formalin, processed through graded alcohol, cleared with xylene, and embedded in paraffin. Sections of paraffinzied tissues (5 μm) were stained with hematoxylin and eosin (H&E). In addition, selected sections were stained with Masson’s trichrome stain to better characterize collagen deposition and fibrosis. For Masson’s trichrome staining, paraffin-embedded tissue sections (4–5 μm thick) were deparaffinized in xylene, rehydrated through graded ethanol solutions, and rinsed in distilled water. Sections were stained with Weigert’s iron hematoxylin for nuclear visualization, followed by Biebrich scarlet-acid fuchsin solution to stain cytoplasmic elements and muscle fibers. Tissue sections were then differentiated in phosphomolybdic-phosphotungstic acid solution and subsequently stained with aniline blue to identify collagen fibers. After rinsing in acetic acid, sections were dehydrated through graded alcohols, cleared in xylene, and mounted with a permanent mounting medium. Collagen fibers appeared blue, muscle and cytoplasmic elements red, and nuclei black (21). Slides were examined independently in a blinded manner by a board-certified veterinary pathologist to evaluate collagen deposition, cellularity, and associated inflammatory changes.

## Results

### Clinical findings

A total of nine adult dromedary camels (8 males and 1 female) were diagnosed with cutaneous keloid fibroma involving the caudal aspect of the thigh region. The animals ranged in age from 50 to 180 months (mean ± SD: 95 ± 12 months) and represented four breeds, including Asfar (*n* = 5), Shageh (*n* = 2), Wadeh (*n* = 1), and Mejhem (*n* = 1) (Table 1). The duration of the lesions varied from 3 months to 3 years. Grossly, all lesions (9/9, 100%) presented as solitary or bilateral, well-circumscribed, firm cutaneous masses covered by intact skin (Figures 1–4). Lesion size ranged from 4 × 3 × 2.5 cm to 50 × 49 × 47 cm (Table 1). The largest lesion was observed in a male Mejhem camel, extended from the caudal thigh region into the upper portion of the leg (Figure 2). Bilateral lesions were identified in one female Shageh camel (Figure 4B).

Palpation revealed that all masses were non-fluctuant, firm in consistency, and non-painful (9/9, 100%). None of the camels exhibited pain upon manipulation or palpation of the lesion. Clinical examination specifically assessed local inflammatory changes, including increased skin temperature, erythema, edema extending beyond the lesion margins, purulent discharge, and pain on palpation. None of these signs were observed in any case, indicating the absence of clinically active inflammation. Mild restriction of hindlimb movement was observed in camels with larger masses, particularly those located near the hip joint.

Lesions demonstrated a slowly progressive course and were reported by owners to increase gradually in size over time. Functional impairment was observed in camels with larger masses, particularly those located near the hip joint. Mild restriction of hindlimb movement was noted in three animals (3/9, 33.3%), while obvious lameness was detected in two cases (2/9, 22.2%), both of which had large lesions exceeding 20 cm in maximal diameter. No systemic abnormalities such as fever, anorexia, weight loss, or altered behavior were detected during clinical examination.

Historical information obtained from owners revealed a history of previous local trauma, or recurrent superficial skin injury at the affected site in six cases (6/9, 66.7%). In the remaining three camels, no specific predisposing cause could be identified. The lesions were consistently located in areas exposed to repeated mechanical stress and friction during locomotion, particularly near the caudal thigh and hip regions. Detailed clinical characteristics of all cases, including age, breed, sex, lesion duration, lesion size, and anatomical location, are summarized in Table 1.

### Ultrasonographic findings

Ultrasonographic examination revealed that all lesions were composed predominantly of chronic fibrous tissue and exhibited heterogeneous internal echogenicity. The masses contained isoechoic to hyperechoic contents with multiple internal septa and were enclosed by a well-defined thick echogenic capsule (Figure 1B). Correlation with histopathological findings demonstrated that these ultrasonographic features were consistent with dense fibrous connective tissue and abundant collagen deposition characteristic of keloid fibroma. The lesions were generally well circumscribed and clearly demarcated from the surrounding tissues. Irregular echogenic strands and septa were observed extending throughout the masses, producing a heterogeneous internal architecture. Variations in echogenicity were observed among cases; some lesions exhibited a relatively homogeneous echogenic pattern, whereas others displayed mixed echogenic regions, reflecting differences in the distribution and density of fibrous tissue within the lesions.

Posterior acoustic enhancement was not observed in any case, supporting the predominantly solid nature of the masses. No distinct anechoic or hypoechoic fluid-filled cavities were identified within the lesions. Ultrasonographic evaluation further demonstrated varying degrees of compression and displacement of the surrounding subcutaneous tissues, particularly in larger masses. However, no ultrasonographic evidence of infiltration or extension into the underlying musculature was detected.

In the largest lesions, particularly those located adjacent to the hip joint, mild distortion of neighboring connective tissue planes was observed. These findings corresponded with the mild movement restriction detected clinically in some affected camels. In larger lesions, especially those located close to the hip joint, mild distortion of adjacent connective tissue planes was observed and may have contributed to restricted movement in some animals. No marked vascularization, active abscess formation or extensive fluid accumulation was detected.

Ultrasound-guided aspiration was successfully performed when indicated and enabled collection of representative samples for microbiological examination. Subsequent comparison of the ultrasonographic, surgical, and histopathological findings confirmed that the echogenic septa and capsule visualized ultrasonographically corresponded to dense hyalinized collagen bundles and mature fibrous connective tissue characteristic of keloid fibroma. This correlation supported the value of ultrasonography as a useful adjunctive diagnostic tool for characterizing the extent and internal architecture of these lesions prior to surgical intervention.

### Microbiological findings

Microbiological examination of samples collected from all nine lesions yielded consistent bacteriological findings. Direct Gram-stained smears revealed numerous Gram-positive cocci arranged predominantly in irregular grape-like clusters. Aerobic culture on sheep blood agar produced small (approximately 2–3 mm in diameter), circular, smooth, glistening, opaque, creamy-white colonies that were slightly sticky in consistency and exhibited weak hemolysis. No mixed bacterial growth was detected in any sample.

Preliminary phenotypic characterization demonstrated that all isolates were catalase-positive and coagulase-negative. Subsequent identification using the VITEK® 2 Compact automated system confirmed the isolates as *Staphylococcus warneri* in all cases, with an identification probability of ≥98% and an excellent confidence level (Table 2).

**Table 2:** Individual culture and antimicrobial susceptibility results of isolates recovered from cutaneous keloid fibromas in dromedary camels should be provided as supplementary material.

Case No.	Sample Type	Gram Stain	Bacterial Isolate (VITEK 2)	Identification Probability (%)	Levofloxacin	Moxifloxacin	Tigecycline	Tetracycline	Benzyl Penicillin	Erythromycin
1	Tissue/Pus	Gram-positive cocci in clusters	*Staphylococcus warneri*	≥98	S	S	S	S	R	R
2	Tissue/Pus	Gram-positive cocci in clusters	*Staphylococcus warneri*	≥98	S	S	S	S	R	R
3	Tissue/Pus	Gram-positive cocci in clusters	*Staphylococcus warneri*	≥98	S	S	S	S	R	R
4	Tissue/Pus	Gram-positive cocci in clusters	*Staphylococcus warneri*	≥98	S	S	S	S	R	R
5	Tissue/Pus	Gram-positive cocci in clusters	*Staphylococcus warneri*	≥98	S	S	S	S	R	R
6	Tissue/Pus	Gram-positive cocci in clusters	*Staphylococcus warneri*	≥98	S	S	S	S	R	R
7	Tissue/Pus	Gram-positive cocci in clusters	*Staphylococcus warneri*	≥98	S	S	S	S	R	R
8	Tissue/Pus	Gram-positive cocci in clusters	*Staphylococcus warneri*	≥98	S	S	S	S	R	R
9	Tissue/Pus	Gram-positive cocci in clusters	*Staphylococcus warneri*	≥98	S	S	S	S	R	R

S, sensitive; R, resistant.

Antimicrobial susceptibility testing revealed a largely consistent susceptibility profile among the recovered isolates. All isolates were susceptible to levofloxacin, moxifloxacin, tigecycline, and tetracycline, indicating favorable activity of these antimicrobial agents against the recovered organism. In contrast, resistance to benzyl penicillin and erythromycin was consistently identified. The complete culture and antimicrobial susceptibility results for each camel are presented in Table 2.

The recovery of *S. warneri* from all examined lesions suggests a potential role for this opportunistic pathogen as a secondary bacterial colonizer of chronic keloid fibromas. Nevertheless, the relatively low inflammatory response observed clinically and histopathologically indicates that bacterial colonization was unlikely to be the primary cause of lesion development and was more likely associated with the chronic nature of the lesions.

### Histopathological findings

All nine lesions affected the dermis and extended into the subcutis. The lesions were located on the caudal aspect of the thigh, predominantly near the hip joint. Grossly, the masses were nodular and measured between 4 × 3 × 2.5 cm and 50 × 49 × 47 cm, with a mean maximal diameter of approximately 18.1 ± 15.1 cm × 14.6 ± 14.6 cm × 11.7 ± 13.5 cm. The overlying epidermis remained intact in all cases (9/9, 100%). Microscopically, all lesions (9/9, 100%) were composed predominantly of dense fibrous connective tissue characterized by abundant bundles of thick hyalinized collagen fibers (Figure 5A). Sparse fusiform fibrocytes were distributed throughout the collagen bundles in all cases (Figures 5B,C). Minimal inflammatory cell infiltration was observed, consisting of occasional macrophages and lymphocytes located primarily around blood vessels in 3/9 cases (33.3%) (Figure 5D). The central regions of the lesions consistently exhibited lower cellularity and denser collagen deposition compared with the peripheral regions. Toward the lesion margins, the dense hyalinized collagen bundles gradually merged with thinner and more fibrillar collagen fibers (Figure 5D). In 6/9 cases (66.7%), the central portion of the lesion was composed almost entirely of densely packed hyalinized collagen fibers extending into the subepidermal layer (Figure 6A), resulting in compression of adjacent skin adnexal structures, including sweat glands and hair follicles (Figure 6B). Neovascularization and granulation tissue were not observed in any lesion (0/9, 0%). The predominant histological component in all cases consisted of mature hyalinized collagen fibers with scant fibrocytes (Figures 6C,D), consistent with the diagnosis of keloid fibroma. Clinical history revealed evidence of previous trauma, or recurrent skin injury at the affected site in 6/9 camels (66.7%), whereas no identifiable predisposing cause was reported in the remaining three animals. These findings support a possible association between chronic tissue injury and the development of keloid fibroma.

## Discussion

Cutaneous keloid fibroma in dromedary camels in the present research exhibited characteristic features of chronic fibro-proliferative lesions, likely representing a sequela of previous tissue injury and prolonged wound remodeling rather than active inflammation. Clinically, the swellings were firm, well-circumscribed, and non-painful, with no clinical evidence of local heat, erythema, edema, or purulent discharge. These findings are consistent with previous reports describing keloidal and fibrotic lesions as chronic processes characterized primarily by excessive collagen deposition and fibrosis. The presence of a thick fibrous capsule and dense collagenous tissue further supports the chronic nature of these lesions (3–5, 22).

Although sex-related differences have been reported in several surgical and dermatological conditions in camels, the present data are insufficient to establish a definitive association between sex and the development of keloid fibroma (3). The predominance of male camels (8/9, 88.9%) observed in the present study should be interpreted cautiously because of the limited number of animals examined. Additional epidemiological studies involving larger populations are required to investigate potential demographic risk factors.

The affected camels were mature animals ranging from 5 to 15 years of age. The prolonged duration of these lesions, varying from 3 months to 3 years, suggests that keloid fibroma develops gradually over time and remains clinically stable for extended periods. However, because the present research lacked an unaffected comparison group, no conclusions regarding age- or weight-related predisposition can be drawn. Ultrasonographically, the lesions demonstrated heterogeneous echogenicity, internal septations, and a well-defined echogenic capsule. The ultrasonographic appearance corresponded closely with the histopathological findings of dense hyalinized collagen bundles and mature fibrous connective tissue. Similar ultrasonographic characteristics have been reported in chronic fibrotic soft-tissue lesions in camels and other large animals (3, 5). The absence of fluid-filled cavities and marked vascularization further supported the predominantly fibrous nature of the lesions.

Surgical excision resulted in favorable clinical outcomes in all nine cases, with satisfactory wound healing and no recurrence detected during the six-month follow-up period. Complete removal of the fibrous capsule was considered important to minimize the possibility of recurrence. Although the number of cases was limited, these findings support surgical excision as an effective treatment option for localized keloid fibroma in camels. Although no recurrence was detected during the six-month follow-up period in any of the nine camels, the relatively short duration of follow-up limits definitive assessment of long-term recurrence risk. Keloidal and keloid-like fibro-proliferative lesions in other species may recur months to years after surgical excision. Therefore, the favorable outcomes observed in the present study should be interpreted as short-term results, and further longitudinal studies with extended follow-up are necessary to determine the long-term prognosis and recurrence rate of cutaneous keloid fibroma in camels. True keloids are generally considered unique to humans; however, keloid-like fibro-proliferative lesions have been described in veterinary species, particularly dogs, and share several histopathological features with human keloids (3, 23–25). In the present research, the lesions consisted of abundant hyalinized collagen bundles with sparse fibrocytes and minimal inflammatory cell infiltration. Only occasional macrophages and lymphocytes were observed around blood vessels in a minority of cases, indicating limited inflammatory involvement. These findings support the interpretation that camel keloid fibromas are chronic fibro-proliferative lesions rather than actively inflammatory processes.

The isolation of *Staphylococcus warneri* from all examined lesions is noteworthy. Because *S. warneri* is recognized as an opportunistic skin commensal, its recovery from these lesions likely reflects secondary bacterial colonization of chronic fibrotic tissue rather than a primary etiological role in lesion development (26, 27). This interpretation is supported by the minimal inflammatory changes observed clinically and histopathologically.

Recent reports have confirmed the occurrence of keloid fibroma among cutaneous and subcutaneous mass lesions in dromedary camels. El-Shafaey *et al.* (2026) (5) identified keloid fibroma in 23.91% of camel cutaneous and subcutaneous masses, whereas Abu Damir et al. (2025) (6) reported that fibromatous lesions accounted for approximately 11% of tumor-like masses in camels. These findings demonstrate that keloid fibroma is an established, although relatively uncommon, clinical entity in dromedary camels rather than a previously undocumented condition.

Histologically, keloid fibromas were characterized by abundant thick hyalinized collagen fibers and low cellularity. These features clearly distinguished them from exuberant granulation tissue, which is typically characterized by abundant fibroblasts, neovascularization, and inflammatory cells (28, 29). In contrast, none of the camel lesions examined in the present study demonstrated granulation tissue or significant neovascularization. Furthermore, unlike conventional fibromas reported in camels, which are composed of interlacing bundles of fibroblasts and fibrocytes (30–32), the lesions described herein were dominated by mature hyalinized collagen fibers containing only sparse fibrocytes. These findings support the diagnosis of keloid fibroma and emphasize its distinction from other fibrous proliferative lesions.

The precise pathogenesis of keloid fibroma in dromedary camels remains incompletely understood. Chronic tissue injury and prolonged wound remodeling are likely important contributing factors; however, the potential roles of local immune regulation, cytokine-mediated fibroblast activation, and lymphatic drainage abnormalities have not been investigated in camel lesions. In human keloids, dysregulated inflammatory signaling and persistent fibroblast activation are recognized drivers of excessive collagen deposition. Comparable mechanisms may contribute to the development of keloid fibroma in camels, but this hypothesis requires experimental validation. Because the present study was designed as a descriptive study and did not include immunohistochemical or molecular analyses, conclusions regarding immune or lymphatic involvement cannot be drawn. Future studies should investigate the local immune microenvironment and lymphatic architecture of these lesions to improve understanding of their pathogenesis. Based on the gross, histopathological, and clinical findings, the primary pathogenesis of these lesions appears to involve trauma followed by excessive fibroblast proliferation and collagen deposition, resulting in keloidal fibroma formation. In contrast, the isolation of *Staphylococcus warneri* should be interpreted as a secondary complication rather than the principal etiological factor. As an opportunistic coagulase-negative *Staphylococcus* sp., this organism may colonize chronically damaged tissues and contribute to persistent local inflammation, abscess formation, and delayed healing. Therefore, the bacteriological findings likely represent secondary colonization of pre-existing fibro-proliferative lesions rather than evidence that bacterial infection initiated the pathological process (16, 23, 24, 28).

The development of keloidal fibroma in the present cases may reflect a dysregulated fibro-proliferative response associated with chronic local inflammation. Recent morphological, cytological, and immunohistochemical investigations have demonstrated that dromedary lymphoid organs possess distinctive structural and cellular characteristics that influence regional immune responses. The compartmentalized architecture of camel lymph nodes and the specialized cellular composition of lymphoid tissues may contribute to the localization and containment of chronic inflammatory stimuli through enhanced encapsulation rather than complete lesion clearance. Such prolonged immune stimulation could promote excessive collagen deposition and fibroblast proliferation, ultimately contributing to the formation of keloid-like fibrous masses. These observations support the concept that camelid-specific immunological mechanisms may influence the evolution of chronic inflammatory and fibro-proliferative lesions (33–35).

A limitation of the present study is that lesion characterization relied primarily on conventional linear measurements, which may not fully represent the complex three-dimensional morphology of keloidal fibromas. Although tumor length, width, and estimated volume provided useful descriptive data, these measurements cannot adequately capture shape variation, surface irregularities, or spatial growth patterns. Future investigations employing two-dimensional (2D) or three-dimensional (3D) geometric morphometric methods, combined with advanced imaging modalities, could provide more comprehensive quantitative assessments of lesion morphology and improve comparisons among cases and disease stages.

## Conclusion

Cutaneous keloid fibroma in the thigh region of dromedary camels can be reliably diagnosed through the integration of clinical, ultrasonographic, microbiological, and histopathological findings. This multimodal approach allows accurate lesion characterization and supports appropriate therapeutic decision-making. Histologically, these lesions are characterized by dense hyalinized collagen bundles with minimal inflammatory cell infiltration, supporting their classification as chronic fibro-proliferative lesions. Surgical excision with complete removal of the fibrous tissue resulted in favorable healing outcomes in all cases, with no evidence of recurrence during the six-month follow-up period. Early recognition and appropriate surgical management may help prevent lesion enlargement and associated functional impairment, thereby improving animal welfare and clinical outcomes. However, because this report represents a limited cases number from a single institution, further studies involving larger populations of camels are warranted to better define the epidemiology, pathogenesis, and long-term outcomes of this condition. Complete surgical excision resulted in favorable short-term clinical outcomes in the present cases. However, because postoperative monitoring was limited to six months, the absence of observed recurrence should not be interpreted as proof of permanent cure. Further studies involving larger case numbers and extended follow-up periods are required to clarify the long-term prognosis of camel keloidal fibromas.

## Data Availability

The original data supporting the conclusions of this article will be made available by the authors, without undue reservation, upon reasonable request to the corresponding author.
